# Genetic polymorphisms of HLA-DP and isolated anti-HBc are important subsets of occult hepatitis B infection in Indonesian blood donors: a case-control study

**DOI:** 10.1186/s12985-017-0865-7

**Published:** 2017-10-23

**Authors:** Yan Mardian, Yoshihiko Yano, Widya Wasityastuti, Neneng Ratnasari, Yujiao Liang, Wahyu Aristyaning Putri, Teguh Triyono, Yoshitake Hayashi

**Affiliations:** 1 Division of Infectious Disease Pathology, Department of Microbiology and Infectious Disease, Kobe University Graduate School of Medicine, 7-5-1 Chuo-ku, Kobe, 650-0017 Japan; 2 Division of Gastroenterology, Department of Internal Medicine, Kobe University Graduate School of Medicine, 7-5-1 Chuo-ku, Kobe, 650-0017 Japan; 3 Department of Physiology, Faculty of Medicine, Gadjah Mada University, Kesehatan Street No. 1, Sekip, Yogyakarta, 55281 Indonesia; 4 Division of Gastroenterohepatology, Department of Internal Medicine, Dr. Sardjito Hospital, Faculty of Medicine, Gadjah Mada University/ Dr. Sardjito Hospital, Kesehatan Street No. 1, Sekip, Yogyakarta, 55281 Indonesia; 5 Division of Molecular Medicine & Medical Genetics, Department of Pathology, Kobe University Graduate School of Medicine, 7-5-1 Chuo-ku, Kobe, 650-0017 Japan; 6 Department of Clinical Pathology, Dr. Sardjito Hospital, Faculty of Medicine, Gadjah Mada University/ Dr. Sardjito Hospital, Kesehatan Street No. 1, Sekip, Yogyakarta, 55281 Indonesia

**Keywords:** OBI, HLA-DP SNPs, Indonesian blood donor

## Abstract

**Background:**

Occult hepatitis B infection (OBI) is defined as the presence of hepatitis B virus (HBV) DNA in the serum and/or liver in HBsAg-negative individuals. OBI is associated with the risk of viral transmission, especially in developing countries, and with progressive liver disease and reactivation in immunosuppressive patients. The objective of this study was to evaluate the relation of OBI to HLA-DP single nucleotide polymorphisms (SNPs) encoding antigen-binding sites for the immune response to HBV infection. As HLA-DP variants affect the mRNA expression of HLA-DPA1 and HLA-DPB1 in the liver, we hypothesised that high levels of HLA-DPA1 and HLA-DPB1 expression favour OBI development.

**Methods:**

The study enrolled 456 Indonesian healthy blood donors (HBsAg negative). OBI was defined as the presence of HBV-DNA in at least two of four open reading frames (ORFs) of the HBV genome detected by nested PCR. SNPs in HLA-DPA1 (rs3077) and HLA-DPB1 (rs3135021, rs9277535, and rs2281388) were genotyped using real-time Taqman® genotyping assays.

**Results:**

Of 122 samples positive for anti-HBs and/or anti-HBc, 17 were determined as OBI. The minor allele in rs3077 was significantly correlated with OBI [odds ratio (OR) = 3.87, 95% confidence interval (CI) = 1.58–9.49, *p* = 0.0015]. The prevalence of the minor allele (T) was significantly higher in subjects with OBI than in those without (59% and 33%, respectively). The combination of haplotype markers (TGA for rs3077–rs3135021–rs9277535) was associated with increased risk of OBI (OR = 4.90, 95%CI = 1.12–21.52 *p* = 0.038). The prevalence of OBI was highest in the isolated anti-HBc group among the three seropositive categories: anti-HBs <500 mIU/ml, anti-HBs ≥500 mIU/ml, and isolated anti-HBc (29.41%, *p* = 0.014).

**Conclusion:**

Genetic variants of HLA-DP and the presence of anti-HBc are important predictors of OBI in Indonesian blood donors.

**Trial registration:**

Ref: KE/FK/194/EC; registered 01 March 2013. Continuing approval Ref: KE/FK/536/EC; registered 12 May 2014.

**Electronic supplementary material:**

The online version of this article (10.1186/s12985-017-0865-7) contains supplementary material, which is available to authorized users.

## Background

Occult hepatitis B virus (HBV) infection (OBI) is defined as the presence of HBV DNA in the serum and/or liver of HBsAg-negative individuals. Since its discovery in 1963 and its subsequent association with HBV infection, hepatitis B surface antigen (HBsAg) remains the main serological marker in routine laboratory tests for the detection of infection. It greatly reduced HBV transmission due to blood transfusions, as transfusion of HBsAg positive blood is prohibited [1, 2]; however, numerous scientific papers have highlighted the presence of HBV infection in individuals who test negative for HBsAg and have detectable HBV DNA in the liver or blood [1–3]. The prevalence of OBI, which has been reported worldwide, varies greatly across the globe, with higher rates reported in Asia; however, cases have also been reported in low HBV endemic areas [4]. Indonesia has the third-highest prevalence of HBV infection worldwide, with a moderate-to-high hepatitis B endemicity that affects 242 million people [5, 6].

Although it has been recognized since the 70s, OBI became a topic of interest in hepatology research in 1999, when a study published in The New England Journal of Medicine identified a large series of HBsAg-negative patients with chronic liver disease (CLD) who were positive for HBV genomes by testing liver biopsy specimens [7]. That study highlighted the clinical importance of OBI, which can promote or accelerate the progression of hepatitis C virus (HCV)-related chronic hepatitis to cirrhosis. In addition, it provided a new perspective on virological issues by showing that OBI viruses have no genetic mutations capable of preventing viral replication or HBsAg synthesis [8].

Significant advances in our understanding of the molecular mechanism underlying OBI have been made in the past decade [4]. Sequence variations in HBV genomes, including S gene variants (S-escape mutants), can result in conformational changes of HBsAg that render the protein undetectable by commercially available detection kits [4, 8]. In addition, in a small number of cases, OBI is linked to HBV mutants with defective replication activity or synthesis of S proteins, which cause the lack of detectable HBsAg despite the presence of HBV genomes [9, 10]. However, in most cases, OBI genomes are replication-competent viruses with genetic heterogeneity comparable to that of HBV isolates from individuals with “overt” (HBsAg-positive) infection. OBI status is therefore thought to result from a strong suppression of HBV replication and gene expression involving different mechanisms [8]; however, knowledge of the factors involved in the suppression of viral activity leading to the induction of OBI remains limited. Understanding these factors is important as it may provide new insights into HBV virology and reduce the probability of transmission.

HBV has indirect cytopathic properties, indicating that the host immune response is involved in regulating viral activity, including viral replication and disease progression [6]. One important finding of genome-wide association studies is that polymorphisms in the Human Leucocyte Antigen (HLA) DPA1/DPB1 genes, which are located on the short arm of chromosome 6, influence HBV infection [11, 12]. HLA-DP molecules belonging to HLA class II play an important role in adaptive host-immune responses, particularly in antigen presentation to CD4+ T helper cells [13, 14]. A recent study in Indonesia confirmed that HLA-DPA1 and HLA-DPB1 variants are associated with outcomes of HBV infection such as susceptibility, persistent infection, and disease progression [6]; however, to date, the effect of HLA-DP variants on OBI detection has not been investigated in an Indonesian population. It would be interesting to investigate the possible association of variations in HLA-DP loci with the suppression of HBV replication resulting in OBI, which is immune-mediated. The aim of the present study was to investigate the profiles and genetic influence of HLA-DPA1/DPB1 single nucleotide polymorphisms (SNPs) on the detection of OBI in the Indonesian population. This study focused on one SNP in HLA-DPA1 (rs3077) and three SNPs in HLA-DPB1 (rs3135021, rs9277535, and rs2281388). In addition, this study also investigated the prevalence of OBI and association between HBV antibodies and OBI findings in Indonesian blood donors.

## Methods

### Participants and protocol

The present study recruited Indonesian blood donors from Dr. Sardjito Hospital, Yogyakarta, Indonesia, between April 2013 and November 2014. A total of 456 healthy participants of Javanese ethnicity were enrolled from the blood donation unit. During their first visit, all subjects provided verbal informed consent for the storage of blood samples for further studies. The present study also recruited 121 Japanese subjects from Kobe University Hospital for comparison of the linkage disequilibrium (LD) pattern between the two populations.

All participants had to meet the following inclusion criteria: normal physical examination and tested negative for hepatitis B surface antigen (HBsAg), hepatitis C virus (HCV), and human immunodeficiency virus (HIV). The study protocol conformed to the ethical guidelines of the 1975 Declaration of Helsinki and was approved by the Medical and Health Research Ethics Committee (MHREC) Faculty of Medicine, Gadjah Mada University (Trial registration: Ref: KE/FK/194/EC [01 March 2013] Additional file 1, Ref: KE/FK/536/EC [12 May 2014]) Additional file 2. All subjects provided written informed consent before enrolment.

### Serological tests

All subjects were screened for HBsAg, anti-HCV antibodies, and anti-HIV antibodies using automated chemiluminescent enzyme immunoassays on an Architect analyzer (Abbott Laboratories, IL, USA). Subjects testing negative were further examined for antibodies to the hepatitis B surface antigen (anti-HBs) and hepatitis B core antibodies (anti-HBc) using chemiluminescence immunoassays (CLIA) (Architect AUSAB, Abbott Japan). Anti-HBs and anti-HBc titers were considered positive at threshold values of ≥ 10.0 mIU/ml and ≥1.0 s/CO, respectively.

### DNA extraction and SNP genotyping

For HBV DNA extraction and SNP genotype determination, peripheral venous blood was drawn and collected into EDTA blood tubes. HBV DNA and genomic DNA were extracted from 200 μl of serum and buffy coats using the QIAamp DNA Blood Mini Kit; Qiagen, Hilden, Germany), in accordance with the manufacturer’s instructions.

Genotyping of each subject for HLA-DPA1 (rs3077) and HLA-DPB1 (rs3135021, rs9277535, and rs2281388) variants was performed using the Allelic Discrimination Assay on a 7500 Real-Time PCR system with TaqMan® Genotyping Master Mix (Applied Biosystems, Foster City, CA, USA). The SNP rs3077 (located in the 3′ untranslated region [UTR] of HLA-DPA1) was selected because it is representative of the DPA1 haplotype block, and rs9277535 (in the 3′ UTR of HLA-DPB1) and rs2281388 (in the downstream region of HLA-DPB1) were selected because they were functionally different SNPs in the DPB1 haplotype block, as determined using Haploview 4.2 software (available at http://hapmap.ncbi.nlm.nih.gov/) [15]. rs3135021 (in intron 1 of HLA-DPB1) was also selected because it did not belong to any haplotype block. SNPs were genotyped as previously described with specific primers [15] and FAM and VIC-labelled probes provided by Sigma-Aldrich (Hokkaido, Japan) in compliance with recommended protocol [16]. All four SNPs were successfully genotyped at rates of >97.5%. Quality control for each assay was performed using samples with known genotypes obtained by direct sequencing.

### HBV amplification and quantification of HBV DNA

HBV DNA was detected using a previously described nested PCR method with primers targeting the S, polymerase (Pol), precore-core, and X regions of the HBV genome, with slight modifications. The primers targeting the four Open Reading Frames (ORFs) were used as previously described [17]. The conditions for the first and second rounds of PCR were 95 °C for 10 min, followed by 40 cycles of 95 °C for 30 s, 58 °C for 30 s, 72 °C for 1 min, and the final extension at 72 °C for 10 min. Appropriate controls were included in each PCR reaction. OBI positivity was based on the detection of HBV DNA by nested PCR in at least two regions out of four ORFs in anti-HBs and/or anti-HBc positive samples (seropositive).

Quantification of HBV DNA was performed using the TaqMan PCR Assay (lower limit of detection, 2.1 log copies/ml) in a Roche-LightCycler® 96 Real-Time PCR System.

### Direct sequencing and genotype determination

The amplified product from the second round of PCR was purified using ExoSAP-IT (USB Corporation, Cleveland, OH, USA) according to the manufacturer’s instructions. The PCR products were sequenced using the BigDye Terminator version 3.1 cycle sequencing kit on an ABI Prism 3100-Avant genetic analyser (Applied Biosystems, Foster City, CA, USA). The nucleotide sequences obtained from direct sequencing and the reference sequences retrieved from GenBank were aligned with Clustal X Software [18].

Phylogenetic trees were constructed using the neighbour-joining method, and bootstrap resampling was performed 1000 times. The analyses were performed using Molecular Evolutionary Genetics Analysis (MEGA) software.

### Statistical analysis

The Hardy–Weinberg equilibrium (HWE) of the genotype distributions and the LD of the SNPs were examined using Haploview software v4.2 (http://www.broadinstitute.org/haploview/haploview) [19]. Differences in categorical variables and continuous variables were compared using the Pearson χ2 test and Student’s t test, respectively. The genotype frequency represented the frequency of the major homozygous (MM), heterozygous (Mm), and minor homozygous (mm) alleles corresponding to each SNP (rs3077 corresponds to C/T, rs3135021 corresponds to G/A, rs9277535 corresponds to G/A, and rs2281388 corresponds to C/T).

Genetic associations (genotype-based, allele-based, and haplotype-based) were tested using the χ2 test or Fisher’s exact test. Three different genetic association models were generated: the additive genetic model [minor allele (m) versus (vs.) major allele (M)], in which each copy of a minor allele modifies the risk in an additive manner, resulting in the homozygous minor (mm) having a two-fold higher risk than the heterozygote (Mm) [comparing 2 mm + Mm (Ad) vs. MM]; the dominant genetic model [heterozygote and minor homozygote (Mm + mm) vs. major homozygote (MM)]; and the recessive genetic model [minor homozygote (mm) vs. major homozygote and heterozygote (MM + Mm)].

Logistic regression was performed to compare cases and control groups, and all odds ratios (ORs), 95% confidence intervals (CIs) and *p* values were adjusted for age and sex. Haplotype frequency was estimated using a two-stage iterative method, the Expectation Maximization algorithm.

Statistical analyses were performed using SPSS version 22 (IBM Corporation, Armonk, NY, USA), whereas allele-based and haplotype-based genetic models were applied using SNPStats web tools (http://bioinfo.iconcologia.net/SNPstats.) [20]. Statistical significance was defined by *P* values of <0.05.

## Results

### Characteristics of subjects

The characteristics of seropositive subjects are summarized in Table 1. Among 456 healthy participants (380 men and 76 women; mean age 29.95 ± 9.78 years) who were HBV surface antigen (HBsAg) negative, 122 cases (26.75%) were detected by anti-HBs and/or anti-HBc antibody and were designated as seropositive. Among them, 17 subjects were diagnosed with OBI and compared with subjects without OBI (Non-OBI). Subjects in the OBI group were older than those in the Non-OBI group, although the difference was not statistically significant (35.50 ± 13.52 years versus 30.92 ± 10.73 years, *P* = 0.136). No significant differences in gender and average anti-HBs and anti-HBc titers were observed between the two groups.

**Table 1:** Clinical demographics of study participants (122 seropositive subjects)

Demographic	OBI	NON-OBI	*P* Value ^a^
No. of seropositive subjects	17	105	.. .
Age, years (mean ± SD)	35.50 ± 13.52	30.92 ± 10.73	0.136
No. of male subjects (%)	14 (82.35)	87 (82.85)	0.959
Anti-HBs, mIU/mL (mean ± SEM)	339.1 ± 132.2	451.7 ± 103.6	0.670
Anti-HBc, s/CO (mean ± SEM)	4.054 ± 1.031	2.885 ± 0.350	0.227
*Categories of seropositive subjects*			
a. Anti-HBs <500 mIU/mL (%) b. Anti-HBs ≥500 mIU/mL (%) c. Isolated anti-HBc ^b^ (%)	6 (7.60) 6 (23.08) 5 (29.41)	73 (92.40) 20 (76.92) 12 (70.59)	**0.014**

*Abbreviations*: *SD* standard deviation, *SEM* standard error of mean

^a^Bold indicates statistically significant

^b^Isolated anti-HBc is defined by samples that were positive for anti-HBc (≥1.0 s/CO) but negative for anti-HBs (<10.0 mIU/mL)

### OBI findings in seropositive samples

To determine the effect of HBV-specific humoral immune responses on OBI detection, the study subjects were divided into three categories as follows: (1) anti-HBs <500 mIU/mL (regardless of anti-HBc status), (2) anti-HBs ≥500 mIU/mL (regardless of anti-HBc status), and (3) isolated anti-HBc (only anti-HBc was detected as positive). The proportion of OBI cases differed significantly between the three categories. The highest OBI detection rate was observed in the isolated anti-HBc group (29.41%, *P* = 0.014) (Table 1). OBI was more frequent in the isolated anti-Hbc group than in the non-isolated anti-Hbc group (29.41% vs. 9.30%, *P* = 0.049) (data not shown).

The profiles of the 17 OBI cases are summarized in Table 2. All OBI cases had a positive result of nested PCR in the HBV X Region (HBx). The precore/core, surface, and polymerase genes were detected in 29.41%, 64.70%, and 47.05% of cases, respectively. Serum HBV was detected in all OBI samples, mainly at <2.9 log copy/mL (151 IU/mL). Only one OBI case had 3.3 log copy/mL (379.3 IU/mL), which was still within the range of the low threshold of HBV DNA quantification.

**Table 2:** Baseline characteristics of OBI in seropositive samples

No	Sample Code	Hepatitis serology titer		Sex	Age (years)	Nested PCR result (HBV ORF)*				HBV titer (log copy/ml)	Genotype/Sub Genotype
		Anti-HBs (mIU/mL)	Anti-HBc (s/CO)			Precore/Core Gene	Surface Gene	Polymerase Gene	X Gene		
1	A026	119.87	4.15	Male	45	+	+	–	+	<2.9 (+)	C1
2	A084	558.91	0.12	Male	56	+	+	–	+	2.9	B
3	A138	0.4	11.04	Male	51	–	–	+	+	<2.4 (+)	B3
4	S032	504.67	0.07	Male	23	+	–		+	<2.4 (+)	B3
5	S049	2084.76	0.09	Female	32	+	+	+	+	<2.4 (+)	B3
6	U064	0.72	7.97	Male	27	–	+	–	+	<2.4 (+)	B3
7	U075	132.41	0.39	Male	22	–	–	+	+	2.4	B
8	U110	14.81	0.09	Male	23	–	+	+	+	<2.4 (+)	B
9	U132	33.2	1.94	Male	50	–	–	+	+	<2.4 (+)	B3
10	U152	560.56	0.8	Male	24	–	+	–	+	<2.4 (+)	B7
11	U154	29.96	0.14	Female	24	–	+	+	+	<2.4 (+)	B3
12	U169	852.06	7.61	Male	24	+	–	–	+	<2.4 (+)	B3
13	U250	15.91	0.21	Male	18	–	+	+	+	<2.4 (+)	B3
14	U258	850.22	8.97	Male	50	–	+	–	+	<2.4 (+)	B7
15	U282	1.41	9.02	Male	31	–	–	+	+	<2.4 (+)	B3
16	U313	3.61	6.06	Female	46	–	+	–	+	<2.4 (+)	C1
17	U338	1.09	10.25	Male	54	–	+	–	+	3.3	B3

^*^OBI was determined by the detection of HBV-DNA using nested PCR in at least two regions out of four ORF

### Evaluation of the hardy–Weinberg equilibrium

The association of four SNPs in the HLA-DP gene with the detection of OBI is shown in Table 3. Four SNPs were analyzed in this case-control study, one in HLA-DPA1 (rs3077) and three in HLA-DPB1 (rs3135021, rs9277535, and rs2281388). The genotype frequencies of three polymorphisms (rs3077, rs3135021, and rs9277535) conformed to HWE (*P* > 0.05 each), indicating no significant differences between the observed and expected frequencies of each genotype in these three groups; however, rs2281388 deviated from HWE because the genotype distribution was highly skewed. Therefore, rs2281388 was excluded from further statistical genetic analysis.

**Table 3:** Associations between HLA-DP variants with the detection of OBI

Gene, chromosome position^a^, allele (major/minor)	SNPs ID^b^	NON-OBI			MAF (%)	OBI			MAF (%)	Testing of mode of inheritance	NON-OBI vs OBI	
		Genotype frequency (%)				Genotype frequency (%)						
											*P*-value^c^	OR (95%CI)^d^
		CC/GG	CT/GA	TT/AA		CC/GG	CT/GA	TT/AA				
HLA-DPA1, 33,033,022, C/T	rs3077	43 (41)	53 (51)	8 (8)	33	2 (12)	10 (59)	5 (29)	59	Additive (2 T/T-C/T vs C/C)	**0.0015**	**3.87 (1.58–9.49)**
										Dominant (C/C vs C/T-T/T)	**0.0067**	**6.12 (1.30–28.85)**
										Recessive (C/C-C/T vs T/T)	**0.016**	**5.56 (1.45–21.29)**
HLA-DPB1, 33,045,558, G/A	rs3135021	54 (53)	37 (37)	10 (10)	28	9 (53)	7 (41)	1 (6)	26	Additive (2A/A-G/A vs G/G)	0.8	0.90 (0.41–2.01)
										Dominant (G/G vs G/A-A/A)	0.99	1.01 (0.35–2.87)
										Recessive (G/G-G/A vs A/A)	0.54	0.54 (0.06–4.53)
HLA-DPB1, 33,054,861, G/A	rs9277535	39 (38)	50 (48)	15 (14)	38	4 (24)	11 (65)	2 (12)	44	Additive (2A/A-G/A vs G/G)	0.56	1.26 (0.58–2.72)
										Dominant (G/G vs G/A-A/A)	0.28	1.89 (0.57–6.33)
										Recessive (G/G-G/A vs A/A)	0.76	0.79 (0.16–3.89)
HLA-DPB1, 33,060,118, C/T	rs2281388	70 (67)	24 (23)	11 (1)	22	13 (76)	3 (18)	1 (6)	15	Additive (2 T/T-C/T vs C/C)	rs2281388 deviated from Hardy–Weinberg Equilibrium because the genotype distribution was skewed	
										Dominant (C/C vs C/T-T/T)		
										Recessive (C/C-C/T vs T/T)		

^a,b^: SNP identification numbers and positions (http://www.ncbi.nlm.nih.gov/) based on Human Genome Assembly build GRCh37.p17;. ^c,d^: Logistic regression analyses were adjusted for gender and age. Bold values indicate statistically significant

The correlation between HLA-DP variants and OBI detection in seropositive subjects was investigated by comparing the alleles of the OBI and Non-OBI groups. The minor allelic frequencies (MAFs) of rs3077 (T), rs3135201 (A), and rs9277535 (A) were 33%, 28%, and 38%, respectively, in the Non-OBI group, and 59%, 26%, and 44%, respectively, in the OBI group (Table 3). HLA-DP rs3077 was associated with OBI detection in seropositive subjects, with the minor allele “T” associated with increased probability of OBI (additive genetic model: *P* = 0.0015, OR: 3.87, 95% CI: 1.58–9.49; dominant genetic model: *P* = 0.0067, OR: 6.12, 95% CI: 1.30–28.85; recessive genetic models: *P* = 0.016, OR: 5.56, 95% CI: 1.45–21.29). HLA-DPB1 rs3135021 and rs9277535 were not associated with OBI detection in any of the genetic models tested (all *P* > 0.05); however, the frequency of GA and AA genotypes of HLA-DPB1 rs9277535 was higher in the OBI group than in the Non-OBI group (dominant genetic model: *P* = 0.28, OR: 1.89, 95% CI: 0.57–6.33), although the difference was not statistically significant after adjusting for age and gender (Table 3).

**Fig. 1:**
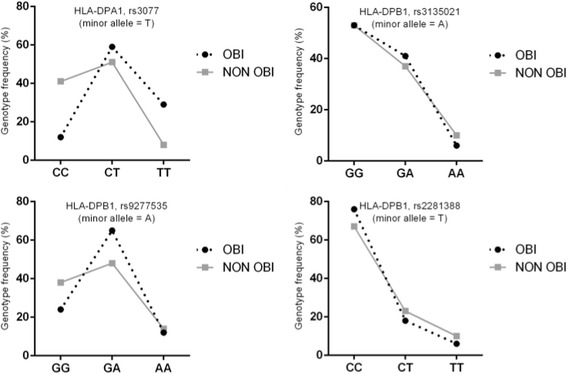
Genotypic distribution of the four polymorphisms. Genotyping of each subject for HLA-DPA1 (rs3077) and HLA-DPB1 (rs3135021, rs9277535, rs2281388) variants at rates of >97.5%

The genotypic distribution of the four polymorphisms is depicted in Fig. 1. The major homozygous genotypes were more frequent in the Non-OBI group than in the OBI group in rs3077 and rs9277535: CC (Non-OBI 41% vs. OBI 12%) and GG (Non-OBI 38% versus OBI 24%), respectively. Similarly, in rs3077 the minor homozygous genotype was more frequent in the OBI group than in the Non-OBI group at 29% vs. 8%, respectively; however, that trend was not observed in rs9277535, in which the genotype frequency of the minor homozygous allele was comparable between the OBI and Non-OBI groups at 12% and 14%, respectively. The other two HLA-DPB1 SNPs examined in this study (rs3135021 and rs2281388) did not show significant differences in genotype distribution between the OBI and Non-OBI groups.

### Linkage disequilibrium and haplotype analysis of multiple SNPs

Haplotypes were constructed based on the three HLA-DP polymorphisms in Hardy–Weinberg equilibrium (*P* > 0.05). The association between the combination of three SNPs and OBI detection in seropositive subjects is shown in Table 4. Based on the HLA-DP haplotype constructed from SNPs rs3077 (minor allele “T”), rs3135021 (minor allele “A”), and rs9277535 (minor allele “A”), comparison of the target haplotypes with the remaining haplotype combinations showed that haplotype block TGA was significantly associated with the detection of OBI in seropositive subjects (*P* = 0.038, OR: 4.90, 95% CI: 1.12–21.52). All other haplotype combinations showed no significant association with OBI detection.

**Table 4:** Haplotype association model

Haplotype (rs3077–rs3135021–rs9277535)	Frequency (%)		NON-OBI vs OBI	
	NON-OBI	OBI	*P*-value^a^	OR (95%CI) ^b^
C-G-G	0.3764	0.1983	–	1.00
T-G-A	0.1782	0.3564	**0.038**	**4.90 (1.12–21.52)**
C-A-G	0.1677	0.1772	0.51	1.74 (0.34–8.80)
C-G-A	0.1051	0.0363	0.51	0.46 (0.05–4.55)
T-A-A	0.0785	0.0485	0.93	1.12 (0.10–12.84)
T-G-G	0.0598	0.1443	0.091	5.31 (0.78–36.12)
T-A-G	0.0131	0.0391	0.17	10.91 (0.38–315.08)

^a,b^: Logistic regression analyses were adjusted for gender and age. Bold values indicate statistically significant

**Fig. 2:**
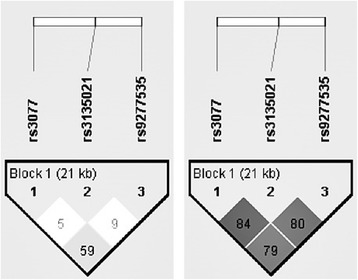
Linkage disequilibrium (LD) map of the three HLA-DP SNPs. The LD values represent the D’ values in the Indonesian population (left) and the Japanese population (right)

The three SNPs assessed are located within an LD block spanning >21 kb. The pairwise LD data for the SNPs examined in this Indonesian population are shown in Fig. 2. This study also compared this LD block between the Indonesian and Japanese subjects included in the study. In Indonesian subjects, the three SNPs in HWE (rs3077, rs3135021, and rs9277535) were in weak LD with each other, with the D’ values ranging from 0.05 to 0.59; however, the D’ value between rs3077 and rs9277 was significantly higher than that between the other two (D’ value = 0.59). Overall, these D’ values were stronger in the Japanese population (0.79–0.84).

### Direct sequencing of HBV DNA and HBV genotyping

**Fig. 3:**
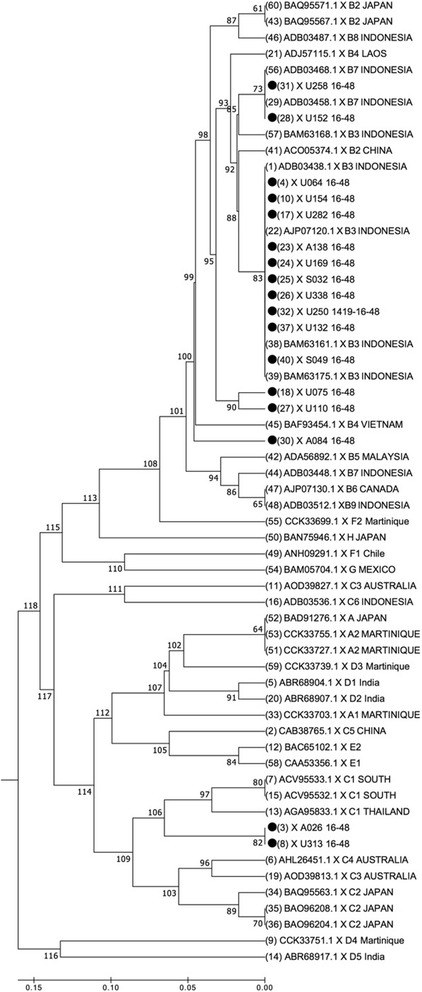
Neighbour-joining phylogenetic tree for HBV strains in OBI subjects (indicated with solid circles). The phylogenetic analysis was performed based on 101 bp of the partial X region. The bootstrap support for the consensus tree, inferred from 1000 replicates, is indicated for each branch. The evolutionary distances were computed using the maximum composite likelihood method

Since only the X gene was positive in the nested PCR results for the 17 OBI samples, we performed direct sequencing from the amplified product of the second round of X gene PCR for determination of the HBV genotype (Fig. 3).

The phylogenetic tree revealed that most of the samples had the HBV genotype B (88.2%), with subgenotype B3 as the most common (64.7%), and the remaining samples showing subgenotype B7 (11.8%) and subgenotype C1 (11.8%).

Due partly to difficulties of DNA sequencing with low levels of viral DNA, only a partial sequence of the surface gene was detected in one sample, and the previously reported OBI amino acid changes that affect the predicted antigenicity (T123A, M133 L, T143 M, and T126I) were not detected in our sample [21].

## Discussion

The introduction of universal vaccination has strongly reduced vertical transmission in many countries; however, horizontal transmission is also an important route of HBV infection. In particular, blood transfusion from healthy donors with OBI is associated with the risk of horizontal transmission [22]. OBI is not only difficult to diagnose, but is also associated with a variety of clinical conditions. Several reports have indicated that OBI has similar infectivity and pathogenicity in the development of fulminant hepatitis, liver cirrhosis, and hepatocellular carcinoma (HCC) with non-occult infection, and that it may affect the safety of blood transfusions and orthotopic liver transplantation (OLT) [3]. The role of OBI in chronic hepatitis C virus (HCV) infection is perhaps the most extensively studied, as OBI can promote or accelerate the progression of HCV-related CLD, as minimal lesions produced by the immune system in response to OBI may contribute to disease progression [23, 24]. Evidence validated by meta-analyses highlights the pro-oncogenic role of OBI and the risk of viral reactivation in immunocompromised OBI patients, which may lead to the (re)development of HBV-related liver disease [4, 8].

This study showed that the isolated anti-HBc serological status defined an important subset of OBI (Table 1). This result is consistent with that of previous studies in which isolated anti-HBc was identified as a significant serological marker of OBI [25, 26]. Isolated anti-HBc positivity refers to the detection of anti-HBc antibody without the usual accompanying markers (i.e., HBsAg if the infection is chronic and anti-HBs if the infection is resolved) [26]. The core antigen is a potent immunogen that elicits a strong and specific antibody response that is present during acute infection and usually persists for life; therefore, the presence of anti-HBc in the absence of any other marker is compatible with resolved or past hepatitis B infection [27, 28]. Several studies have highlighted the strong correlation between OBI and anti-HBc status [26, 29]. One investigation reported that of 16 patients with detectable serum HBV DNA at enrolment, 10 (63%) were positive for anti-HBc, whereas only 17 (42%) of 40 patients without serum HBV DNA tested positive for anti-HBc [30]. Similarly, studies show that the rate of HBV DNA is significantly high in anti-HBc–positive but anti-HBs–negative individuals, with a frequency of up to 60% in populations highly exposed to the virus [31]. The fact that OBI is more frequent in subjects positive for anti-HBc but negative for anti-HBs is presumably because these subjects lack the neutralizing effect of anti-HBs [32]; consequently, in the absence of hepatitis B DNA testing, isolated anti-HBc is an indicator of an important subset of OBI [25, 26]. Our study highlighted the superiority of anti-HBc over other HBV markers in predicting latent HBV infection in apparently healthy individuals and indicated that implementation of anti-HBc screening would improve the safety of blood donation.

In the present study, most OBI genomes were genotype B3. This is in accordance with previous studies showing that in Indonesia the most common HBV genotype is HBV B3 (HBV/B3), followed by HBV/C1, especially in Java island [21, 33].

The mechanisms underlying the low HBV DNA levels in the absence of detectable HBsAg in OBI remain unclear; however, both host and viral factors are important for viral replication suppression and for maintaining the control of HBV infection [34]. Several studies have suggested that host factors play a major role in the induction and maintenance of the occult status of HBV infection [24], as implied by an in vitro study showing that once the viruses are removed from the host’s liver microenvironment, the survival abilities of OBI isolates, in terms of replication, transcription, and protein synthesis, can be fully restored [35]. Further evidence was provided by a large-scale study that demonstrated the presence of potent and multi-specific HBV-specific T cell responses in OBI subjects. In that study, HBV-specific Th1 responses were quantitatively stronger in OBI than in inactive carriers and were similar or even higher against HBV antigens than those in patients with previous HBV resolution [34]. Therefore, the OBI phenomenon not only indicates the lack of complete clearance of HBV, but also reflects the ability of the host immune system to control leftover viruses in the liver after clinical resolution of disease through an efficient T-cell mediated response [34, 36].

The present study hypothesized that high levels of HLA-DPA1 and HLA-DPB1 expression will favour OBI development. HLA-DP is a heterodimer of specialized glycoproteins that deliver foreign peptides to the surface of the cell, and it belongs to a major isotype of HLA class II. It consists of two chains, the α (DPA) and β (DPB) chains, and plays a vital role in adaptive immunity [6]. The genes encoding the α and β chains (designated A and B, respectively) are expressed on the surface of antigen presenting cells, whereas in the liver their expression is limited to a small population of Kupffer cells, the resident macrophages of the liver [12]. These molecules encode proteins that are crucial for presenting HBV peptides to CD4^+^ helper T-cells by promoting T-cell allorecognition and peptide binding, resulting in an enhanced immune response and HBV clearance [37, 38]. CD4^+^ T cells also stimulate and preserve CD8^+^ cytotoxic T cells, which directly clear HBV-infected cells. As a consequence, a reduction in HLA-DP expression might interfere with antigen presentation and impair adaptive and cellular immune responses to HBV infection [6].

The present results support a previous theory on the impact of efficient T cell mediated immune responses on OBI induction and maintenance. We have shown that the minor T allele of rs3077 was the prominent allele detected in the OBI seropositive group in all the genetic models analyzed (Table 3). Furthermore, the T/A variants of rs3077 and rs9277535 in the haplotype model were also associated with a higher probability of OBI detection (Table 4). These findings are supported by previous studies that reported the involvement of HLA-DP polymorphisms in regulating antigen presentation, both at the cellular function and gene expression levels [39].

A previous study suggested that HLA-DP variants could influence the level of mRNA expression of HLA-DPA1 and HLA-DPB1 molecules in the liver, whereas other studies showed that a variant in the 3′ untranslated region (UTR) may affect mRNA stability through binding of regulatory factors or regulation by microRNAs [12, 37, 40]. The most convincing and well-investigated SNPs related to HBV infection and clearance, especially in the Asian population, are rs3077 and rs9277535, which are separated by approximately 22 kb and located within the 3′-UTR of HLA-DPA1 and HLA-DPB1, respectively [11, 12, 41–43]. Because these SNPs are not located in the HLA-DP coding region, their biological effects are investigated by indirectly affecting the antigen-binding site through alterations in microRNA-binding sites leading to changes in HLA-DP gene expression [12]. This change could affect the stability and translation of mRNA by altering transcription factor binding. The minor “T” allele of rs3077 and minor “A” allele of rs9277535 are associated with increased mRNA expression of their respective genes [12, 38]. Therefore, high HLA-DPA1 and HLA-DPB1 expression might be more effective for presenting viral antigens to CD4^+^ helper T-cells, which may increase the capacity of the immune response to suppress viral replication [38]. The present study confirmed that rs3077-T and rs9277535-A, which were associated with increased mRNA expression of the HLA-DP gene, may favour a stronger immune response to suppress HBV replication, which leads to OBI.

Wasityastuti et al. confirmed that in the Indonesian population, HLA-DP variants exert a protective effect against HBV infection by reducing the susceptibility to HBV and increasing the spontaneous resolution of HBV infection [6]. This conclusion strengthens the evidence that the outcome of HBV infection could be influenced by the physical binding of HBV-derived peptides and their subsequent recognition by CD4^+^ helper T-cells, which is dependent on HLA-DP polymorphisms [14]; however, to the best of our knowledge, the present study is the first to examine the roles of HLA-DP variants in OBI.

This study reported a weak LD between rs9277535 and rs3077 (D’ = 0.59; Fig. 2), suggesting that the effects of these SNPs are likely to be independent [12]; however, the difference in LD values between the Indonesian and Japanese populations (Fig. 2) may be caused by the number of ethnic groups in Indonesia, which has a more heterogeneous population than Japan [6]. Japan is dominated by Mainland Japanese (98.5%), whereas Indonesia consists of three dominant ethnic groups (Javanese, 40.06%; Sundanese, 15.51%; and Malay, 3.7%) and more than 10 smaller ethnic groups [6, 44]. This multi-ethnic population provides a higher opportunity for mixture and gene flow that contribute to LD reduction [45]; however, this study found no association between HLA-DPA1 rs3135021 and OBI detection, although the haplotype association model indicated that the major allele “G” increased the probability of OBI. This result might be attributable to the sample size and the genetic diversity of the populations. A phylogenetic tree of 54.794 autosomal SNPs in 1928 individuals representing 75 populations shows that the Indonesian clade clustered separately from the Japanese, Korean, Chinese, Taiwanese, and Thai clades with a high bootstrap value. Indonesia, which is located near the equator, has a higher haplotype diversity than other populations of Northern latitudes [6, 46]. Accordingly, HLA-DPB1 rs2281388 deviated from HWE, which could be related to laboratory or genotyping factors, sample stratification, or random evolutionary changes that might lead to unreliable results [6, 47, 48].

## Conclusion

In conclusion, HLA-DP variants were associated with OBI detection in seropositive Indonesian blood donors. The minor allele of rs3077 (T) in the HLA-DPA1 gene was related to an increased risk of OBI. A combination of haplotype markers (TGA for rs3077–rs3135021–rs9277535) was also associated with OBI detection. The HBV serological marker (isolated anti-HBc) may play an important role in predicting latent HBV infection. The results suggest that the combination of SNP genotyping in the HLA-DP gene and HBV serological marker testing may be a valuable screening method in the blood donation setting to prevent OBI transmission through blood donors.

## Additional file

Additional file 1: Ethical Clearance dr Widya 2013 (FIRST). (JPG 215 kb)
Additional file 2: Ethical Clearance dr Widya 2014 (SECOND). (PDF 996 kb)

## Abbreviations

Anti-HBc: Antibody to hepatitis B core antigen
Anti-HBs: Antibody to hepatitis B surface antigen
CIs: Confidence intervals
CLD: Chronic liver disease
CLIA: Chemiluminescence enzyme immunoassays
HBsAg: Hepatitis B surface antigen
HBV: Hepatitis B virus
HCC: Hepatocellular carcinoma
HCV: Hepatitis C virus
HIV: Human Immunodeficiency virus
HLA: Human leukocyte antigen
HLA-DPA: Human leucocyte antigen DP gene alpha chain
HLA-DPB: Human leukocyte antigen DP gene beta chain
HWE: Hardy–Weinberg equilibrium
LD: Linkage Disequilibrium
MAF: Minor allelic frequencies
MEGA: Molecular Evolutionary Genetics Analysis
mRNA: Messenger RNA
OBI: Occult hepatitis B infection
OLT: Orthotopic liver transplantation
ORF: Open reading frame
ORs: Odds ratios
SNP: Single nucleotide polymorphisms
UTR: Untranslated region
