# Supplementary figures and images for: Genetic polymorphisms of HLA-DP and isolated anti-HBc are important subsets of occult hepatitis B infection in Indonesian blood donors: a case-control study
Source: Virol J. 2017 Oct 23;14:201. doi: 10.1186/s12985-017-0865-7 (PMC5654084; doi:10.1186/s12985-017-0865-7)

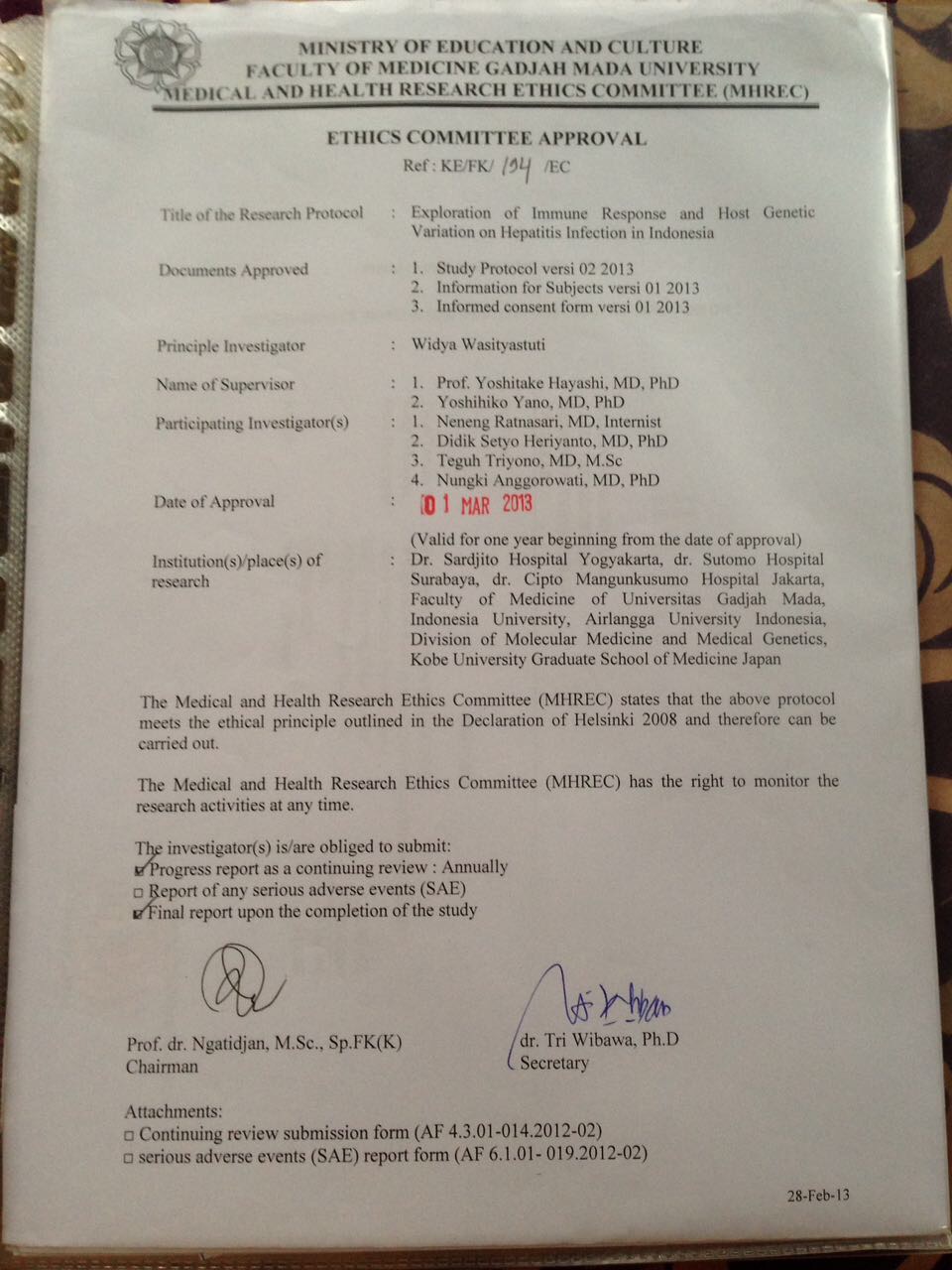

Supplement: Additional file 1: — Ethical Clearance dr Widya 2013 (FIRST). (JPG 215 kb) [file 12985_2017_865_MOESM1_ESM.jpg]
