# Supplementary material for: Genetic polymorphisms of HLA-DP and isolated anti-HBc are important subsets of occult hepatitis B infection in Indonesian blood donors: a case-control study
Source: Virol J. 2017 Oct 23;14:201. doi: 10.1186/s12985-017-0865-7 (PMC5654084; doi:10.1186/s12985-017-0865-7)
Supplement: Additional file 2: — Ethical Clearance dr Widya 2014 (SECOND). (PDF 996 kb) [file 12985_2017_865_MOESM2_ESM.pdf]

# MEDICAL AND HEALTH RESEARCH ETHICS COMMITTEE (MHREC)
# FACULTY OF MEDICINE GADJAH MADA UNIVERSITY
# – DR. SARDJITO GENERAL HOSPITAL

## CONTINUING REVIEW APPROVAL OF APPROVAL

**Ref: KE/FK/194/EC Year 2013**

Ref : KE/FK/536 /EC

| | | |
|---|---|---|
| Title of the Research Protocol | : | Exploration of Immune Response and Host Genetic Variation on Hepatitis Infection in Indonesia |
| Documents Approved | : | 1. Study Protocol versi 2012<br>2. Information for Subjects versi 2012<br>3. Informed consent form versi 2012 |
| Principle Investigator | : | Widya Wasityastuti |
| Name of supervisor | : | 1. Prof. Yoshitake Hayashi, MD, PhD<br>2. Yoshihiko Yano, MD, PhD<br>3. Neneng Ratnasari, MD, M.Sc<br>4. Didik Setyo Heriyanto, MD, PhD<br>5. Teguh Triyono, MD, M.Sc<br>6. Nungki Anggorowati, MD, PhD |
| Date of Approval | : | 12 MAY 2014<br>(Valid for one year beginning from the date of approval) |
| Institution(s)/place(s) of research | : | Dr. Sardjito Hospital Yogyakarta, dr. Sutomo Hospital Surabaya and dr. Cipto Mangunkusumo Hospital Jakarta |

The Medical and Health Research Ethics Committee (MHREC) states that the above protocol meets the ethical principle outlined in the Declaration of Helsinki 2008 and therefore can be carried out.

The Medical and Health Research Ethics Committee (MHREC) has the right to monitor the research activities at any time.

The investigator(s) is/are obliged to submit:
- □ Progress report as a continuing review : Annually
- □ Report of any serious adverse events (SAE)
- ☑ Final report upon the completion of the study

| | |
|---|---|
| Prof.dr.Mohammad Hakimi, Sp.OG (K),Ph.D<br>Chairman | dr. Madarina Julia, Sp.A(K).,MPH.,Ph.D<br>Secretary |

Attachments:
- □ Continuing review submission form (AF 4.3.01-014.2012-02)
- □ Serious adverse events (SAE) report form (AF 6.1.01- 019.2012-02)
